# Low-Frequency Stimulation Prevents Kindling-Induced Impairment through the Activation of the Endocannabinoid System

**DOI:** 10.1155/2021/5526780

**Published:** 2021-06-16

**Authors:** Sina Khajei, Khadijeh Esmaeilpour, Javad Mirnajafi-Zadeh, Vahid Sheibani, Soheila Rezakhani, Yaser Masoumi-Ardakani

**Affiliations:** ^1^Neuroscience Research Center, Institute of Neuropharmacology, Kerman University of Medical Sciences, Kerman, Iran; ^2^Department of Physiology, Faculty of Medical Sciences, Tarbiat Modares University, Tehran, Iran; ^3^Physiology Research Center, Institute of Basic and Clinical Physiology Sciences, Kerman University of Medical Sciences, Kerman, Iran

## Abstract

**Background:**

Cannabinoid system affects memory and has anticonvulsant effects in epileptic models. In the current study, the role of cannabinoid 1 (CB1) receptors was investigated in amelioration of the effects of low-frequency stimulation (LFS) on learning and memory impairments in kindled rats.

**Methods:**

Electrical stimulation of the hippocampal CA1 area was employed to kindle the animals. LFS was applied to the CA1 area in four trials following the last kindling stimulation. One group of animals received intraperitoneal injection of AM251 (0.1 *μ*g/rat), a CB1 receptor antagonist, before the LFS application. Similarly, CB1 agonist WIN55-212-2 (WIN) was administrated to another group prior to LFS. The Morris water maze (MWM) and the novel object recognition (NOR) tests were executed 48 h after the last kindling stimulation to assess learning and memory.

**Results:**

Applying LFS in the kindled+LFS group restored learning and memory impairments in the kindled rats. There was a significant difference between the kindled and the kindled+LFS groups in learning and memory. The application of AM251 reduced the LFS effects significantly. Adversely, WIN acted similarly to LFS and alleviated learning and memory deficits in the kindled+WIN group. In addition, WIN did not counteract the LFS enhancing effects in the KLFS+WIN group.

**Conclusions:**

Improving effects of LFS on learning and memory impairments are mediated through the activation of the endocannabinoid (ECB) system.

## 1. Background

Epilepsy is a common chronic neurological disease that has many characteristics such as recurrent unpredictable seizures, brain damages, and cognitive-psychiatric comorbidities. The most common form of epilepsy in humans is temporal lobe epilepsy [1, 2]. Electrical kindling is one of the frequently used models to induce epilepsy in rats in order to study various antiepileptic drugs [2]. This model is timely controlled. Therefore, stimulations can be stopped at any moment to observe the after discharge signals [3]. Kindling effect is described as the occurrence of after discharges and behavioral seizures due to repetitive electrical stimulation of the limbic brain structures [3, 4].

The limbic areas, especially the hippocampus, are damaged by kindled seizures [5, 6]. Deficits in the hippocampus include problems associated with learning new information, information recovery difficulties, and spatial learning and memory problems [7, 8]. The improving effect of low-frequency stimulation (LFS) on kindling-induced impairments in rats is evident in many studies [8–10]. LFS has anticonvulsant effects on epileptic seizures in kindled rats such as reduced behavioral seizure stages, after discharge duration, stage-5 seizure duration, and increased latency to stage-4 seizure [11]. Our previous experiment showed that LFS application in fully kindled animals had improving effects on spatial learning and memory [8].

Many substances or drugs may alter the kindling-induced impairments through inhibitory or excitatory synaptic functions. They may also potentiate or depotentiate synaptic plasticity. For example, cannabinoids (CBs) are reported to influence synaptic plasticity [12]. Endogenous cannabinoid ligands and cannabinoid 1 (CB1) receptor agonists have anticonvulsant effects in epileptic models [13]. The endocannabinoid (ECB) system influences synaptic plasticity as a regulator of some pathways or as a transducing mechanism retrograding synaptic messages [12]. Endocannabinoids (ECBs) activate presynaptic CB1 receptors of inhibitory and excitatory neurons; therefore, the release of the neurotransmitters is reduced [14].

Activation of the ECB system by inhibition of ECB degrading enzymes provides the opportunity to activate the CB1 receptors selectively at the site of the excessive neuronal activation to prevent epilepsy development [14]. WIN55-212-2 (WIN), which is a CB1 agonist, protected against cognitive impairment in rats [15]. Immediate microinjection of AM281, a CB1 receptor antagonist, after kindling stimulation reduced the following LFS effect on potentiation. Activation of CB1 receptors via ECBs may mediate the inhibitory effects of LFS on kindled seizures [16]. Cannabinoids alter plasticity and memory in the hippocampus [12, 17, 18]. The effectiveness of LFS in the treatment of epilepsy has led to the renewed research efforts to find its anticonvulsant mechanisms in the optimization of treatment paradigms. When the anticonvulsant effects of ECB receptors and their role in synaptic plasticity are considered, it seems that there is an interaction between CB1 receptors and LFS. Therefore, the current study is aimed at investigating the role of CB1 in the amelioration of the effects of LFS on learning and memory impairments induced by kindled seizures in rats.

## 2. Methods

### 2.1. Animals

Male Wistar rats (250–300 g) were delivered from Kerman animal farm (Kerman, Iran). There were 8 experimental groups, each containing 7 rats (56 rats in total). Four rats were housed in each cage, and they had free access to food and water. The temperature and the light-dark cycles were controlled (23 ± 1°C; lights on 07:00–19:00). The Ethics Committee of Kerman Neuroscience Research Center (ethics code: KNRC-95-48) approved all the protocols and treatments. After the experiments, rats were sacrificed under carbon dioxide euthanasia in compliance with the euthanasia recommendations stated in the “Guide for the Care and Use of Laboratory Animals.”

### 2.2. Surgery, Kindling, and LFS Procedure

We used the same procedure as our previous work [8, 9]. The rats were anesthetized using a combination of ketamine (100 mg/kg) and xylazine (12 mg/kg). The injections were intraperitoneal (I.P.). Tripolar configuration electrode was implanted in the hippocampal CA1 area (AP: -2.3 mm; ML: 1.7 mm; and DV: 2.6 mm below the dura). Two of the poles were used for bipolar stimulation, and the other pole was used for recording. A Teflon-coated stainless steel wire with 127 *μ*m diameter was used (AM-Systems, USA) to make the electrodes. The tips of the electrodes were exposed using a sharp blade. A screw at the left skull surface was used as the ground and the differential electrode. The electrodes were connected to a head stage and were fixed to the skull using two screws and dental acrylic. At the end of the procedure, electrode placements were confirmed histologically.

The recovery time for each rat was at least 7 days. Then, a 3 s pulse train consisting of 1 ms 50 Hz monophasic pulses with the amplitude of 10 *μ*A was applied to determine the after discharge (AD) threshold. After a 5 min interval, the amplitude of the train was raised by 10 *μ*A. The raising procedure was continued until an AD, lasting for at least 5 seconds, was recorded. When the AD threshold intensity was determined, every rat was stimulated daily with the determined intensity in a semirapid kindling procedure until three consecutive stage-5 seizures (fully kindled state) were elicited, according to the Racine scale [19]. These daily stimulations were comprised of 12 electrical stimulations that were applied every 10 minutes.

**Figure 1:**
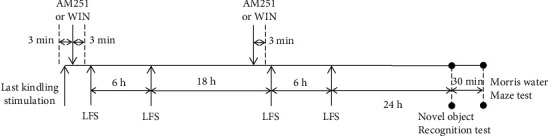
Time-line diagram showing the protocol used for AM251 or WIN55-212-2 (WIN) injections and low-frequency stimulation (LFS) application in the KLFS+AM251 or KLFS+WIN groups.

Both kindling and LFS were applied through the hippocampal CA1 electrode. Fully kindled animals received LFS according to the following: 4 trains, each containing 200 pulses at 1 Hz frequency and at 5-minute intervals, were applied in four trials following the last kindling stimulation. The first and the second LFS were applied immediately and 6 hours following the last kindling stimulation, respectively. The third and the fourth LFS were applied the next day similarly, i.e., at a 6 h interval (Figure 1).

### 2.3. Drug Administration

AM251, which is a selective CB1 receptor antagonist (Sigma-Aldrich Co. LLC, St. Louis, MO, USA), was dissolved in dimethyl sulfoxide (DMSO) and further diluted in saline (0.9% NaCl). DMSO concentration was under 10%. This DMSO-saline solution was used as the vehicle. AM251 was administered 3 minutes before the first LFS stimulation each day. The dose of AM251 (0.1 *μ*g/rat) was selected based on the previous studies [20, 21]. WIN55-212-2 (WIN) was used as the CB1 agonist in our study. Its dose (5 mg/kg) was chosen based on the previous studies [22], and the preparation process was similar to that of AM251.

### 2.4. Experimental Design

Animals were divided into 8 groups: kindled, kindled+AM251, kindled+LFS (KLFS), KLFS+AM251, kindled+WIN, KLFS+WIN, LFS, and control. The animals in the kindled group received kindling stimulations to achieve a fully kindled state. In the KLFS group, fully kindled rats received LFS as explained previously. The control group did not receive any kind of stimulation. In the LFS group, the animals received only LFS. In the kindled+AM251 group, fully kindled rats received AM251. In the KLFS+AM251 group, fully kindled rats received LFS and a dose of AM251 that was injected 3 minutes before the first LFS stimulation each day. In the kindled+WIN group, the kindled animals received a dose of WIN agonist (5 mg/kg) per day. Moreover, each day, a dose of WIN agonist was injected intraperitoneally into the animals of the KLFS+WIN group 3 minutes before the first LFS stimulation (Figure 1).

### 2.5. Morris Water Maze (MWM) Test

The same procedure as our previous work was used to assess kindling, LFS, and AM251 effects on spatial learning and memory of the rats in the Morris water maze (MWM) [8]. The MWM was a black circular swimming pool (diameter: 160 cm, height: 80 cm, and water depth: 40 cm) filled with water. The MWM was divided into four equal quadrants. Some visual cues were placed around it. A hidden submerged black square platform (each edge: 10 cm and depth: 1.5 cm beneath the water surface) was placed in the middle of the target quadrant. The behavior of the rats was recorded via a camera (above the center of the pool) and a recording system (Noldus Ethovision® system 7.1). The MWM included three phases: the acquisition phase, the probe test (two hours after the acquisition phase), and the visible platform test. Each phase was performed in three blocks. Every block was comprised of four trials. In the spatial acquisition phase (the first phase), the rats were released into the water while their faces were toward the wall of the quadrant. They were allowed to find the hidden submerged platform in 60 seconds (4 trials with intervals of 20 seconds). After 30 minutes, they started from a different randomly chosen block (4 trials were performed in each block). Every rat was released from 4 different releasing points. After finding the platform, the rats could rest for 20 seconds. Every trial, the escape latency to find the hidden platform, path length, and velocity were calculated. In the probe test phase (the second phase), spatial memory retention was evaluated. The platform was removed in this phase. Every rat was allowed to swim for 60 seconds. The percentages of time and distance of swimming in the target quadrant (quadrant 4) were the criteria to assess spatial memory retention. In the last phase, the visible platform test was performed to assess the possibility of interferences among sensory, motor, or motivation functions. In this test, the escaping ability of the rats to find the visible platform was evaluated (the platform was placed 2 cm above the water surface and was distinguishable via an aluminum foil).

### 2.6. Novel Object Recognition (NOR) Test

We used the same procedure as our previous work [8]. The rats had 10 minutes to habituate to an empty plastic cage (60 × 60 × 40 cm) with uniform illumination. In the training session, twenty-four hours after the habituation session, the rats were allowed to explore two identical objects that were always presented at the same locations for 5 minutes. In the 3-minute test session (15 minutes after the training session), memory retention was assessed. In the test phase, familiar and novel objects were placed at the same locations as the training sessions. The new object was the same as the familiar object (material and size) except that its shape was different. The location of the new object was changed pseudorandomly to avoid locational natural preference. The box and the objects were cleaned using 70% ethanol for the new rats that were being tested. The time spent sniffing or touching the objects was defined as the exploration time. A camera recorded the whole procedure. Discrimination ratio (recognition index) in the training and test phases was defined as the ratio of the exploration time for each object to the total exploration time for both objects (multiplied by 100).

### 2.7. Statistical Analysis

Two-way ANOVA and post hoc Tukey's test were used to assess the effects of the interventions (kindling, LFS, CB1 agonist, and CB1 antagonist) on different experimental groups. These methods compared the differences in learning rates among the different experimental groups in our behavioral tests. The data of the memory tests were analyzed using one-way ANOVA. In case of finding statistical significance among the groups, Tukey's post hoc multiple comparison tests were performed to determine which of the means were different in the groups. In the current study, *p* < 0.05 was interpreted as statistically significant.

## 3. Results

### 3.1. The Effects of LFS, Kindling, CB1 Agonist (WIN), and CB1 Antagonist (AM251) on the MWM Test Results

**Figure 2:**
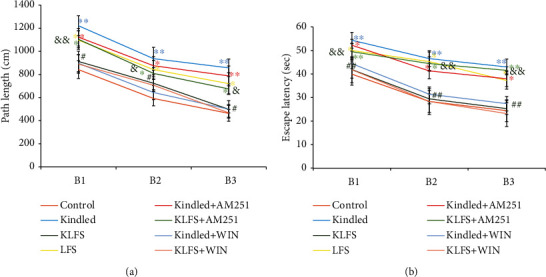
The effects of low-frequency stimulation (LFS), kindling, cannabinoid 1 (CB1) agonist (WIN55-212-2), and CB1 antagonist (AM251) on spatial learning in the Morris water maze (MWM) test. (a) Traveled distance (path length) and (b) escape latency to find the hidden platform in all three blocks. In the spatial acquisition phase of the MWM, every rat was allowed to swim for 60 seconds to find the submerged platform. Increased amounts of traveled distance (path length) and escape latency, in comparison to the control group, indicate spatial learning impairment. Data are shown as mean ± SEM. ^∗^*p* < 0.05 and ^∗∗^*p* < 0.01 (compared to the control group); ^#^*p* < 0.05 and ^##^*p* < 0.01 (compared to the kindled group); ^&^*p* < 0.05 and ^&&^*p* < 0.01 (compared to the KLFS group).

**Figure 3:**
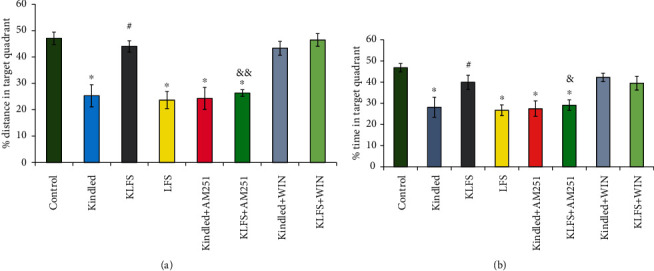
The effects of low-frequency stimulation (LFS), kindling, cannabinoid 1 (CB1) agonist (WIN55-212-2), and CB1 antagonist (AM251) on spatial memory in the Morris water maze (MWM) test. (a) Traveled distance percentage in the target quadrant (Q4) and (b) time percentage in the Q4. In the probe test phase of the MWM, the submerged platform was removed from the Q4. Every rat was allowed to swim for 60 seconds, and its traveled distance and spent time in the Q4 were measured. Decreased amounts, in comparison to the control group, indicate spatial memory impairment. Data are shown as mean ± SEM. ^∗^*p* < 0.05 (compared to the control group); ^#^*p* < 0.05 (compared to the kindled group); ^&^*p* < 0.05 and ^&&^*p* < 0.01 (compared to the KLFS group).

The effects of LFS, kindling, WIN, and AM251 on spatial learning and memory in the MWM test are demonstrated in Figures 2 and 3. The tests were held 48 hours following the last kindling stimulation for all groups (*n* = 7). The increments in the traveled distance and escape latency, as well as the decrements in spent time and distance in the target quadrant, indicate spatial learning and memory impairments, respectively.

Kindling resulted in increased traveled distance (path length) (*F*(7, 48) = 73.85) and escape latency (*F*(7, 48) = 29.50) to find the hidden platform in all three blocks (Figure 2), compared to the control group (*p* < 0.01). LFS alone also resulted in the increased traveled distance and escape latency in the LFS group in comparison to the control group (*p* < 0.05); and the response of the LFS group was similar to that of the kindled group. The LFS and kindling combination resulted in the decreased traveled distance (*p* < 0.05) and escape latency (*p* < 0.01) in all three blocks in the KLFS group in comparison to the kindled group. AM251 in the KLFS+AM251 group increased the path length (*p* < 0.05) and escape latency (*p* < 0.01) in all three blocks in comparison to the control group. AM251 in the KLFS+AM251 group reduced the LFS improvement effects on learning and memory parameters. In addition, during the MWM learning phase, there were differences between the KLFS+AM251 and KLFS groups in path length (block 1: *p* < 0.01, block 2: 0.05, and block 3: 0.05) and escape latency (*p* < 0.01 in the three blocks) factors. WIN (similar to LFS) reversed the kindling impairing effects on learning and memory in the kindled+WIN group compared to the kindled group. In addition, no significant effect was observable in the KLFS+WIN group compared to the KLFS group.

Distance percentage (*F*(7, 48) = 19.32) (Figure 3(a)) and time percentage (*F*(7, 48) = 12.68) (Figure 3(b)) in the target quadrant were deceased after using LFS in the LFS group (*p* < 0.05) or applying kindling stimulations in the kindled group (*p* < 0.05) when compared to the control group. The combination of kindling and LFS, however, increased these parameters in the KLFS group (*p* < 0.05) in comparison to the kindled group. AM251 in the KLFS+AM251 group decreased the traveled distance and spent time in the target quadrant in comparison to the control group (*p* < 0.05). In addition, the time (*p* < 0.05) and distance percentages (*p* < 0.01) in the target quadrant were decreased in the KLFS+AM251 group compared to the KLFS group. AM251 in the KLFS+AM251 group reduced the LFS improvement effect on spatial memory. Administration of AM251 in the kindled animals (kindled+AM251) had no significant effects on spatial learning and memory in comparison to the kindled group. WIN in the kindled+WIN group raised the traveled distance in the target quadrant and the spent time compared to the kindled group. Nevertheless, there were no significant differences between the KLFS and KLFS+WIN groups.

At the start of the study, there were two additional groups in addition to the mentioned groups in “Experimental Design”: the AM251 group and the sham group. The AM251 group received AM251 intraperitoneally, but the rats did not undergo surgery. In the sham group, surgery was performed for the rats. Nonetheless, neither treatments nor any drug administration was applied to this group. The results of these two groups were not significantly different from the control group. Thus, to prevent our figures from becoming overcrowded, we excluded these groups from the figures.

There were no significant differences in swimming speed and the escape latency to find the visible platform among all groups (Table 1). Therefore, kindling, LFS application, and AM251 injection had no effect on visual and motor functions.

### 3.2. The Effects of LFS, Kindling, CB1 Agonist (WIN), and CB1 Antagonist (AM251) on the NOR Test Results

**Figure 4:**
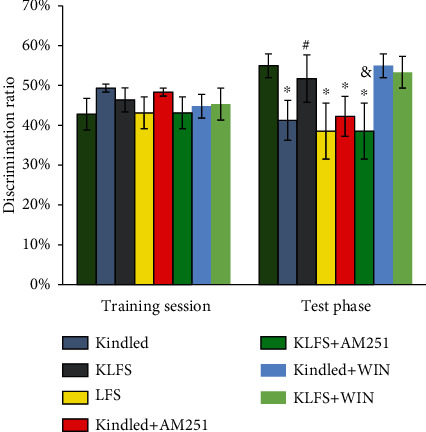
The effects of LFS, kindling, cannabinoid 1 (CB1) agonist (WIN55-212-2), and CB1 antagonist (AM251) on the discrimination ratio in the novel object recognition (NOR) test. In a 5 min training session, the rats were allowed to explore two identical objects that were always situated at the same locations. In a 3 min test session, the time spent for sniffing or touching the objects was defined as the exploration time. Discrimination ratio (recognition index) in training and test phases was defined as the ratio of the exploration time for each object to the total exploration time for both objects. Decreased amounts, in comparison to the control group, indicate memory impairment. Data are shown as mean ± SEM. ^∗^*p* < 0.05 (compared to the control group); ^#^*p* < 0.05 (compared to the kindled group); ^&^*p* < 0.05 (compared to the KLFS group).

In the training phase with two similar objects, all groups spent a similar amount of time in order to explore the objects (Figure 4). In the test phase, the preference to explore the novel object was the indicator of recognition memory. The new object recognition memory was significantly disrupted in the kindled or LFS groups in comparison to the control group (*p* < 0.05) (*F*(7, 48) = 5.88). The kindled rats did not spend a greater amount of time investigating the novel object in relation to the familiar object, and they were not significantly biased toward the novel object. With these animals, the discrimination ratio was significantly lower than that of the control group. The combination of LFS and kindled seizures in the KLFS group increased the discrimination ratio when compared to the kindled group (*p* < 0.05). Injection of AM251 in the KLFS+AM251 group engendered significant impairment effects on spatial learning and recognition memory, and the discrimination ratio was decreased when compared to the control group (*p* < 0.05). Administration of AM251 in the kindled animals (kindled+AM251) had no significant effect on spatial learning and recognition memory in comparison to the kindled group. WIN injection increased the ratio in the kindled+WIN group compared to the kindled group. However, WIN in the KLFS+WIN did not have any significant influence on memory and the discrimination ratio in comparison to the KLFS group.

AM251 decreased the ratio in the KLFS+AM251 group compared to the KLFS group (*p* < 0.05).

## 4. Discussion

Considering anticonvulsant effects of ECB receptors and the effectiveness of LFS in the treatment of epilepsy, we have tried to investigate the role of CB1 in the amelioration of the effects of LFS on learning and memory impairments induced by kindled seizures in rats. In the current study, we have used behavioral methods to determine the role of ECBs. We have studied the effects via behavioral tests. The MWM and NOR tests have been applied to confirm the effects of LFS and to validate the role of ECB receptors.

Our main finding is that the improving effects of LFS on learning and memory impairments are mediated through the activation of the ECB system (CB1), in agreement with other studies [16]. In addition, our results showed that LFS alone or kindling alone resulted in learning and memory impairments. These findings are in agreement with the previous studies [8, 9]. LFS, however, improved learning and memory impairments in the kindled rats. This finding is also consistent with the previous studies [8, 9, 23, 24]. On the one hand, the application of AM251 declined the improving effect of LFS on learning and memory in our study. Mardani et al. (2018) used a similar CB1 antagonist (AM281), and their findings were consistent with our results [16]. In contrast, Barzegar et al. (2015) concluded that AM251 has a memory-improving effect [25]. We suggest that the reason for this contradiction is the use of nonkindled rats in the latter study. On the other hand, administration of WIN resulted in memory-improving effects in the kindled+WIN group in our study, in agreement with previous studies [22]. In addition, there were no significant differences between the KLFS+WIN and KLFS groups. Therefore, it can be concluded that the memory-enhancement mechanism of LFS could be similar to that of the agonist. Based on the results of the KLFS+WIN group alone, it is unclear whether (1) LFS and WIN have independent similar mechanisms of action or (2) LFS influences memory by the mediation of the ECB receptors. The latter suggestion would lead to an indication of a relationship between LFS and ECB receptors. Nonetheless, by considering the results of the antagonist groups, their relationship would be verified.

Cannabinoids impair learning and memory in nonkindled rats. CB1 receptors alter hippocampal synaptic plasticity and spatial memory by regulating dendritic excitability, via the h-current (*I*_h_) regulation, through the hyperpolarization-activated cyclic nucleotide-gated (HCN) channels. Activation of the CB1R-HCN pathway results in impairment in dendritic integration of excitatory inputs. As a result, long-term potentiation (LTP) is formed and spatial memory and learning are impaired [26]. The results of the Barzegar et al. (2015) study indicate that CB1 receptor antagonist partly decreases the effects of glutamatergic receptor antagonist on the passive avoidance test results and improves learning and memory [25]. In contrast, a number of studies have shown that CBs operate by reducing glutamate and GABA releases [17, 27]. CBs, however, improve learning and memory in some situations. CB receptor agonist WIN showed a protective effect on the cognitive impairment induced by chronic cerebral hypoperfusion in rats [15]. The results of our current study also revealed that the use of ECBs had improving effects on learning and memory in the kindled rats. In addition, Abush and Akirav (2010) showed that the CB system has different effects on hippocampal-dependent memory and plasticity. Therefore, it can be concluded that the cannabinoid system activation or deactivation may have impairing or enhancing effects on learning and memory [18].

Izumi and Zorumski (2016) believe that GABA and ECBs mediate depotentiation of Schaffer Collateral synapses, resulting in long-term depression (LTD) and consequently synaptic resetting [28]. The short-term plasticity changes induced by ECBs are altered via depolarization-induced suppression of inhibition of GABAergic transmission and depolarization-induced suppression of excitation of glutamatergic transmission [16]. LFS usage for kindled animals increases the GABAergic currents of the hippocampus [29]. In contrast, based on a number of studies, GABA acts against CB1 receptors and vice versa. LFS increases the glutamatergic currents and reduces GABAergic currents in kindled rats; whereupon, these currents rebound to reach their normal levels [17, 30].

Modulation of cyclic AMP (cAMP) levels by LFS may be the mechanism behind the anticonvulsant effects of LFS. In a study, cAMP levels in the dentate gyrus were increased in the LFS-stimulated kindled rats compared to the control group. Considering that the increment in cAMP levels in the kindled+LFS+vehicle group was significantly lower than the kindled+vehicle group, it can be concluded that LFS prevented kindling-induced cAMP increase. Indeed, LFS had a decreasing effect on cAMP levels in the kindled animals [31]. In addition, other studies have shown that postkindling LFS activated CB1 receptors [16]. Successively, CB1R activation decreases cAMP levels [26]. Thus, based on this sequence, the lessening effect of LFS on cAMP levels is confirmed indirectly.

Synaptic saturation is another explanation for LFS and ECB action mechanisms. Kindling results in synaptic potentiation [32]. Kindling-induced potentiation leads to saturation of all synapses, and consequently, new information storage would become impossible [33]. LFS-induced LTD dampens hippocampal output and suppresses synapses [10]. In addition, CB1 receptors are involved in LTD [34]. Presynaptic CB1 receptor activation results in decreased release of neurotransmitters and consequently LTD [14]. Therefore, it seems that applying LFS (or ECBs) leads to decreased LTP through synaptic potentiation reversal. Finally, synaptic potentiation reversal results in restoring the ability of the synapses to store new information [8, 35].

Neural balance alteration is another hypothesis that explains the relation between synaptic changes and learning and memory increments or decrements. Our results demonstrate that LFS application in healthy rats impairs learning and memory. LFS application in kindled rats, however, improves learning and memory. These results are in agreement with the previous studies [8, 9]. CBs have the same effects. Based on a number of studies, CB receptor activation and deactivation have destructive effects on hippocampal spatial learning and memory [17, 18]. In contrast, CBs have anticonvulsant effects on epilepsy [13]. Our results also showed that the improving effects of LFS on learning and memory impairments are mediated through the activation of the ECB system. Moreover, CB agonists or antagonists upset habitual memory (dorsolateral striatum- (DLS-) dependent memory) because of neural balance disruption. DLS and hippocampus systems compete to control behaviors. Once one is impaired, the other one takes the control of the actions. Therefore, rats or patients are biased to use their in-control memory to carry out the tasks. Therefore, it is concluded that CB agonists or antagonists disrupt healthy memory via triggering neural imbalance [36]. In conclusion, any intervention resulting in restoring the neural balance in hippocampus improves memory, including LFS or ECB applications in kindled rats. LFS or ECB applications in healthy rats, however, impair memory due to triggering the neural balance disruption.

Investigating plasticity was beyond the scope of the current work. Hence, a limitation of our work was that we could not assess LTP. Electrophysiological evaluations would provide valuable information regarding the influences of kindling, LFS, agonists, and antagonists on electrophysiological parameters such as field excitatory postsynaptic potential (fEPSP) and PS amplitude. These parameters are used to study LTP, LTD, or synaptic transmission. Future studies would be needed to investigate LFS mechanisms from the electrophysiological viewpoint. According to our current data, we have shown the effects of LFS and the role of ECB receptors in kindled rats. However, we could not validate all of the mentioned mechanisms described in the discussion section. Therefore, further studies are essential to investigate the possible mechanisms deeply and to find connections among the behavioral, molecular, or other mechanisms.

## 5. Conclusion

In the current study, the effects of LFS and ECBs on kindled seizures were evaluated, and various suggestions were discussed regarding the mechanisms behind the effects. The results of our study indicated that LFS ameliorated kindling-induced learning and memory deficits. The application of antagonist and agonist showed that the improving effects of LFS on learning and memory impairments are mediated through the activation of the ECB system. Considering the discussed mechanisms of the effects of LFS and ECBs, it may be concluded that neural balance alteration would be a promising mechanism explaining the paradoxical effects of LFS and ECBs. Furthermore, the ECB system works through the modulation of synaptic potentials. Hence, future electrophysiological studies would be necessary to clarify the relation between ECBs and LFS action mechanisms.

## Figures and Tables

**Table 1:** Escape latency and swimming speed to find the visible platform in the Morris water maze (MWM) test. These parameters were used to evaluate learning, memory retention, and sensory and motor functions in the rats. Increased amounts of escape latency, in comparison to the control group, indicate spatial learning impairment.

Group	Escape latency (s)	Velocity (cm/s)
Control	23.13 ± 2.23	34.82 ± 0.70
Kindled	25.43 ± 2.45	30.11 ± 1.89
KLFS	28.23 ± 3.03	33.51 ± 2.99
LFS	26.74 ± 2.63	32.57 ± 1.34
Kindled+AM251	24.12 ± 2.98	34.61 ± 2.29
KLFS+AM251	26.76 ± 3.13	31.91 ± 0.89

## Data Availability

The data that support the finding of this study are available from the corresponding author upon reasonable request.

## Abbreviations

CB1: Cannabinoid 1
LFS: Low-frequency stimulation
LTP: Long-term potentiation
LTD: Long-term depression
fEPSP: Field excitatory postsynaptic potential
PS: Population spike
KNRC: Kerman neuroscience research center
MWM: Morris water maze
DLS: Dorsolateral striatum
NOR: Novel object recognition
ECB: Endocannabinoid
DMSO: Dimethyl sulfoxide
WIN: WIN55-212-2.

## Additional Points

*Highlights*. (i) Kindled animals show impairment in learning and memory. (ii) Low-frequency electrical stimulation (LFS) improves learning and memory impairment. (iii) AM251, a cannabinoid 1 (CB1) receptor antagonist, reduces the LFS improvement effects. (iv) WIN55-212-2 protects against learning and memory deficits.

## References

[1] Kandratavicius L., Balista P. A., Lopes-Aguiar C. (2014). Animal models of epilepsy: use and limitations. *Neuropsychiatric Disease and Treatment*.

[2] Pitkänen A., Buckmaster P., Galanopoulou A. S., Moshé S. L. (2017). *Models of Seizures and Epilepsy*.

[3] Gorter J. A., van Vliet E. A., Lopes da Silva F. H. (2016). Which insights have we gained from the kindling and post-status epilepticus models?. *Journal of Neuroscience Methods*.

[4] Goddard G. V., McIntyre D. C., Leech C. K. (1969). A permanent change in brain function resulting from daily electrical stimulation. *Experimental Neurology*.

[5] Sutula T. P. (1990). Progressive neuronal loss induced by kindling: a possible mechanism for mossy fiber synaptic reorganization and hippocampal sclerosis. *Brain Research*.

[6] Pitkänen A., Tuunanen J., Kälviäinen R., Partanen K., Salmenperä T. (1998). Amygdala damage in experimental and human temporal lobe epilepsy. *Epilepsy Research*.

[7] Carreno M., Donaire A., Rocío Sánchez-Carpintero M. D. (2008). Cognitive disorders associated with epilepsy: diagnosis and treatment. *The Neurologist*.

[8] Esmaeilpour K., Sheibani V., Shabani M., Mirnajafi-Zadeh J. (2017). Effect of low frequency electrical stimulation on seizure-induced short-and long-term impairments in learning and memory in rats. *Physiology & Behavior*.

[9] Esmaeilpour K., Sheibani V., Shabani M., Mirnajafi-Zadeh J. (2017). Low frequency electrical stimulation has time dependent improving effect on kindling-induced impairment in long-term potentiation in rats. *Brain research*.

[10] Patel S. (2012). High times for low-frequency stimulation as endocannabinoids engage in hippocampal long-term depression. *Neuropsychopharmacology*.

[11] Gharib A., Sayyahi Z., Komaki A., Barkley V., Sarihi A., Mirnajafi-Zadeh J. (2018). The role of 5-HT_1A_ receptors of hippocampal CA1 region in anticonvulsant effects of low-frequency stimulation in amygdala kindled rats. *Physiology & Behavior*.

[12] Gerdeman G. L., Lovinger D. M. (2003). Emerging roles for endocannabinoids in long-term synaptic plasticity. *British Journal of Pharmacology*.

[13] Andres-Mach M., Zolkowska D., Barcicka-Klosowska B., Haratym-Maj A., Florek-Luszczki M., Luszczki J. J. (2012). Effect of ACEA—a selective cannabinoid CB1 receptor agonist on the protective action of different antiepileptic drugs in the mouse pentylenetetrazole-induced seizure model. *Progress in Neuro-Psychopharmacology and Biological Psychiatry*.

[14] Von Rüden E. L., Bogdanovic R. M., Wotjak C. T., Potschka H. (2015). Inhibition of monoacylglycerol lipase mediates a cannabinoid 1-receptor dependent delay of kindling progression in mice. *Neurobiology of Disease*.

[15] Su S. H., Wang Y. Q., Wu Y. F., Wang D. P., Lin Q., Hai J. (2016). Cannabinoid receptor agonist WIN55,212-2 and fatty acid amide hydrolase inhibitor URB597 may protect against cognitive impairment in rats of chronic cerebral hypoperfusion via PI3K/AKT signaling. *Behavioural Brain Research*.

[16] Mardani P., Oryan S., Sarihi A., Alaei E., Komaki A., Mirnajafi-Zadeh J. (2018). Endocannabinoid CB1 receptors are involved in antiepileptogenic effect of low frequency electrical stimulation during perforant path kindling in rats. *Epilepsy Research*.

[17] Akirav I. (2011). The role of cannabinoids in modulating emotional and non-emotional memory processes in the hippocampus. *Frontiers in Behavioral Neuroscience*.

[18] Abush H., Akirav I. (2010). Cannabinoids modulate hippocampal memory and plasticity. *Hippocampus*.

[19] Racine R. J. (1972). Modification of seizure activity by electrical stimulation: II. Motor seizure. *Electroencephalography and Clinical Neurophysiology*.

[20] Hakimizadeh E., Oryan S., Hajizadeh Moghaddam A., Shamsizadeh A., Roohbakhsh A. (2012). Endocannabinoid system and TRPV1 receptors in the dorsal hippocampus of the rats modulate anxiety-like behaviors. *Iranian Journal of Basic Medical Sciences*.

[21] Roohbakhsh A., Keshavarz S., Hasanein P., Rezvani M. E., Moghaddam A. H. (2009). Role of endocannabinoid system in the ventral hippocampus of rats in the modulation of anxiety-like behaviours. *Basic & Clinical Pharmacology & Toxicology*.

[22] Suleymanova E. M., Shangaraeva V. A., van Rijn C. M., Vinogradova L. V. (2016). The cannabinoid receptor agonist WIN55.212 reduces consequences of status epilepticus in rats. *Neuroscience*.

[23] Gharib A., Komaki A., Manoochehri Khoshinani H. (2019). Intrahippocampal 5-HT_1A_ receptor antagonist inhibits the improving effect of low-frequency stimulation on memory impairment in kindled rats. *Brain Research Bulletin*.

[24] Ahmadirad N., Fathollahi Y., Janahmadi M. (2019). Low-frequency electrical stimulation reduces the impairment in synaptic plasticity following epileptiform activity in rat hippocampal slices through *α*_1_, but not *α*_2_, Adrenergic Receptors. *Neuroscience*.

[25] Barzegar S., Komaki A., Shahidi S., Sarihi A., Mirazi N., Salehi I. (2015). Effects of cannabinoid and glutamate receptor antagonists and their interactions on learning and memory in male rats. *Pharmacology Biochemistry and Behavior*.

[26] Maroso M., Szabo G. G., Kim H. K. (2016). Cannabinoid control of learning and memory through HCN channels. *Neuron*.

[27] Mackie K., Pertwee R. G. (2005). Distribution of cannabinoid receptors in the central and peripheral nervous system. *Cannabinoids*.

[28] Izumi Y., Zorumski C. F. (2016). GABA and endocannabinoids mediate depotentiation of Schaffer collateral synapses induced by stimulation of temperoammonic inputs. *PLoS One*.

[29] Asgari A., Semnanian S., Atapour N. (2016). Low-frequency electrical stimulation enhances the effectiveness of phenobarbital on GABAergic currents in hippocampal slices of kindled rats. *Neuroscience*.

[30] Nazari M., Komaki A., Karamian R., Shahidi S., Sarihi A., Asadbegi M. (2016). The interactive role of CB_1_ and GABA_B_ receptors in hippocampal synaptic plasticity in rats. *Brain Research Bulletin*.

[31] Mohammad-Zadeh M., Mirnajafi-Zadeh J., Fathollahi Y. (2009). The role of adenosine A_1_ receptors in mediating the inhibitory effects of low frequency stimulation of perforant path on kindling acquisition in rats. *Neuroscience*.

[32] Bliss T. V., Collingridge G. L. (1993). A synaptic model of memory: long-term potentiation in the hippocampus. *Nature*.

[33] Chen Y. L., Huang C. C., Hsu K. S. (2001). Time-dependent reversal of long-term potentiation by low-frequency stimulation at the hippocampal mossy fiber–CA3 synapses. *Journal of Neuroscience*.

[34] Izumi Y., Zorumski C. F. (2012). NMDA receptors, mGluR5, and endocannabinoids are involved in a cascade leading to hippocampal long-term depression. *Neuropsychopharmacology*.

[35] Zhang M., Storm D. R., Wang H. (2011). Bidirectional synaptic plasticity and spatial memory flexibility require Ca^2+^-stimulated adenylyl cyclases. *Journal of Neuroscience*.

[36] Goodman J., Packard M. G. (2015). The influence of cannabinoids on learning and memory processes of the dorsal striatum. *Neurobiology of Learning and Memory*.

